# Editorial: Unlocking the value of wastewater: innovative biotechnologies and bioprocesses for resource recovery in a circular economy approach

**DOI:** 10.3389/fmicb.2025.1569498

**Published:** 2025-02-27

**Authors:** Nídia D. Lourenço, Ana B. Lanham, Gilda Carvalho

**Affiliations:** ^1^Associate Laboratory i4HB—Institute for Health and Bioeconomy, NOVA School of Science and Technology, NOVA University Lisbon, Lisboa, Portugal; ^2^UCIBIO—Applied Molecular Biosciences Unit, Department of Chemistry, NOVA School of Science and Technology, NOVA University Lisbon, Lisboa, Portugal; ^3^Department of Bioengineering, iBB—Institute for Bioengineering and Biosciences, Instituto Superior Técnico, Universidade de Lisboa, Lisboa, Portugal; ^4^Associate Laboratory i4HB—Institute for Health and Bioeconomy, Instituto Superior Técnico, Universidade de Lisboa, Lisboa, Portugal; ^5^Australian Centre for Water and Environmental Biotechnology (ACWEB), The University of Queensland, St Lucia, QLD, Australia; ^6^School of Chemical Engineering, University of Queensland, St Lucia, QLD, Australia

**Keywords:** wastewater value, resource recovery, water resource recovery facilities, biological nutrient removal, biopolymers, bioeconomy

In face of the current environmental challenges and the urgent need for sustainable resource management, the valorization of wastewater has become a pivotal strategy toward transitioning into a circular economy. This Research Topic assembles recent research that explores innovative solutions for converting wastewater into value-added resources. The scientific articles in this Research Topic highlight diverse and interdisciplinary approaches taken in order to harness the potential of wastewater, advancing our knowledge on wastewater treatment processes and paving the way for the development of sustainable technologies that contribute to resource recovery and environmental protection.

Nutrient removal and recovery from wastewater is essential for mitigating eutrophication of water bodies and for recycling valuable nutrients that can support sustainable agriculture. Mao et al. have isolated from wastewater a new psychrophile microbial strain, *Acinetobacter kyonggiensis* (AKD4), that is able to perform simultaneous nitrification and denitrification at temperatures as low as 10°C. Lab-scale batch assays showed a total nitrogen removal of 92.45% at 10°C and 87.51% at 30°C, demonstrating its potential for bioremediation in colder climates, while transcriptomics experiments shed light on the various gene expression mechanisms granting tolerance to lower temperatures. On the other hand, Farmer et al. explored the occurrence and functional role of the cyanophycin synthesis gene cphA in activated sludge microbiomes, revealing its widespread presence across many bacteria involved in wastewater treatment. The analysis of over 3,700 metagenome-assembled genomes demonstrated that cphA is actively expressed under different environmental conditions, suggesting that these microorganisms can produce the nitrogen-rich biopolymer cyanophycin. This capacity is a promising opportunity for enhancing nitrogen recovery from municipal wastewater, contributing to nutrient management sustainability. The results highlight the potential for integrating cyanophycin production into existing wastewater treatment processes and advancing the circular nitrogen bioeconomy. Nevertheless, further investigation is needed to elucidate the role and impact of cyanophycin synthesis in wastewater treatment processes, including the dynamics of microbial cphA gene expression and its consequences for nutrient cycling.

Removal and recovery of heavy metals from wastewater is essential for preventing environmental contamination, protecting public health, and enabling the sustainable reuse of treated water in agricultural and industrial applications. The article by Porras-Socias et al. presented a study on vertical subsurface flow constructed wetlands for recycling liquid digestates for agricultural reuse and investigated their treatment performance for the removal of a range of metals and antibiotics, as well as antibiotic resistance genes (ARG). The wetlands were able to effectively remove 82% of the heavy metals tested and 99% of the antibiotics, with immediate decrease of the ARG tested. However, some of the ARG reappeared after 3 months, suggesting adaptation of the community. Further studies are needed to understand and limit the spread of antimicrobial resistance in these biological processes. Gao et al. explored the effect of copper, lead and cadmium on growth and physiological responses of microalga *Dunaliella salina*, revealing its potential for the bioremediation of heavy metal-polluted water. The article details how exposures to these metals adversely impact algal growth and accumulation of key biomolecules, such as polysaccharides and proteins, and demonstrated that *D. salina* is very effective in the absorption of heavy metals under optimized cultivation conditions, with removal efficiencies of 88.9%, 87.2%, and 72.9% for copper, lead, and cadmium, respectively. The associated biochemical reactions were also explored, including changes in antioxidant enzyme activities and malondialdehyde content, hence providing insights into the mechanisms of heavy metal biosorption and its implications for environmental health and sustainability. However, more studies are needed to assess the feasibility of using *D. salina* and other algae species as heavy metal biosorbent in practical applications.

Valorization of wastewater by extraction or production of valuable biomolecules reduces environmental pollution while converting wastes into value-added materials, thus contributing to sustainable resources management and circular economy practices. In light of the recent trends in using photosynthetic systems for carbon neutrality and resource recovery, Wada et al. focused on the downstream processes for purple non-sulfur bacteria cell disruption to recover value-added compounds such as Coenzyme Q10. Overall, PNSB required more intensive protein extraction techniques than were reported to be effective on other single-cell organisms. The NaOH-aided sonication treatment was the most effective protein extraction technique for PNSB biomass, while for CoQ10 extraction, chemical treatment with EDTA was preferable. The study by Chen et al. investigated the extraction and characterization of a water-soluble fraction from the extracellular polymeric substances (EPS) derived from aerobic granular sludge at a full-scale wastewater treatment facility in Zutphen, Netherlands. An alkaline heat method was used for EPS extraction, resulting in a fraction that comprised important biomolecules, such as proteins (26.6%), sugars (21.8%, primarily glucose), and fatty acids. The adhesive properties of this fraction were analyzed, demonstrating shear strength suitable for potential applications as a bio-based adhesive across a wide range of pH values. These findings highlight the potential for valorization of waste sludge, contributing to sustainable practices in wastewater treatment.

Finally, mitigating antibiotic resistance in wastewater is crucial for safeguarding public health, preventing the spread of resistant pathogens, and ensuring the efficacy of antibiotics for future medical treatments. Mannan et al. addressed this pressing issue, identifying and characterizing β-lactamase-producing pathogenic microorganisms in untreated hospital wastewaters to prevent the spread of antibiotic resistance. Out of the 184 isolates, 89 strains were β-lactamase positive with significant resistance to antibiotics such as Ampicillin, Ceftriaxone, Cefuroxime, and Meropenem.

Overall, the articles in this Research Topic highlight the potential of innovative biotechnologies and bioprocesses for unlocking the value of wastewater and aim to inspire further research and innovation in the field of wastewater valorization by advancing our understanding of microbial processes, enhancing resource recovery and developing sustainable materials. The editors hope that the insights and results of this Research Topic will contribute to the ongoing efforts to create a more sustainable future through effective management and use of wastewater resources.

